# Enhancing adoptive cell therapy: future strategies for immune cell radioprotection in neuro-oncology

**DOI:** 10.1038/s41698-025-01059-5

**Published:** 2025-07-29

**Authors:** Abigail J. Groth, Mustafa Khasraw, James D. Byrne, Zachary J. Reitman

**Affiliations:** 1 Department of Radiation Oncology, Duke University School of Medicine, Durham, NC USA; 2 Department of Neurosurgery, Preston Robert Tisch Brain Tumor Center at Duke, Duke University Medical Center, Durham, NC USA; 3 Department of Radiation Oncology, University of Iowa, Iowa City, IA USA; 4 Department of Biomedical Engineering, University of Iowa, Iowa City, IA USA

**Keywords:** CNS cancer, Tumour immunology, Tumour immunology, Radiotherapy

## Abstract

Adoptive cell therapy (ACT), particularly chimeric antigen receptor T cell (CAR T) therapy, has emerged as a promising approach in cancer treatment, demonstrating efficacy in hematological malignancies but facing challenges in brain tumors. The combination of ACT with radiation therapy (RT) offers a potential strategy to enhance therapeutic outcomes, as RT can stimulate immune responses by promoting antigen presentation and T cell recruitment. However, a major hurdle is the radiosensitivity of immune cells, leading to their rapid depletion within the radiation field, which undermines the benefits of this combination. This review explores strategies to increase the radioresistance of immune cells, highlighting the need for innovative radioprotective approaches. We discuss the potential of extremophile-derived molecules, such as the Damage Suppressor protein from tardigrades, as novel radioprotectants that could be integrated into ACT protocols. Furthermore, we address key considerations for clinical trial design, including the sequencing of RT and ACT, dosing parameters, and safety considerations. By bridging insights from extremophile biology and immuno-oncology, this work aims to optimize the efficacy of ACT in the challenging context of brain tumors, paving the way for enhanced treatment strategies in neuro-oncology.

## Introduction

**Fig. 1:**
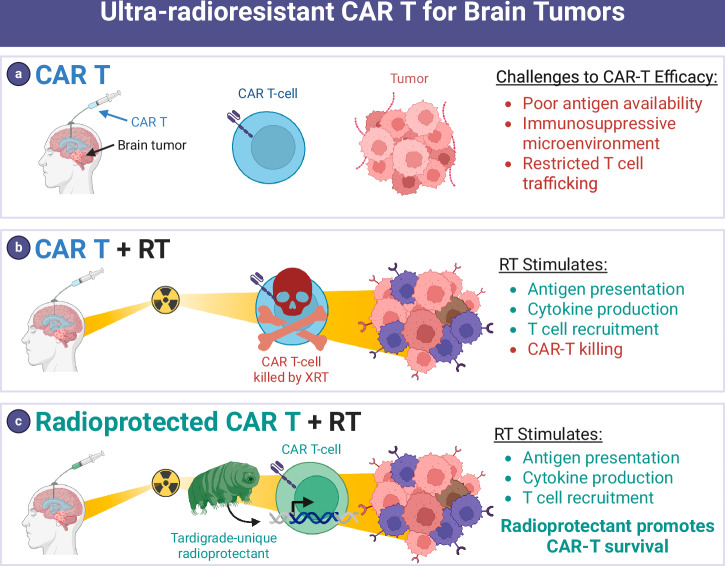
Using an ultra-radioresistant extremophile gene to radioprotect T cells and potentiate ACT. **a** Poor immunogenicity is a key barrier to efficacy for CAR T cells in brain tumors. **b** RT added to CAR T therapy has immunostimulatory effects that may potentiate CAR T therapy. But RT also kills T cells that enter the radiation field which has a counterproductive effect. **c** Radioprotectants such as a gene from the ultra-radioresistant extremophile Tardigrade are expressed in CAR T cells to protect them from RT, allowing immune stimulation from RT to synergize with CAR T.

Adoptive cell therapy (ACT) including chimeric antigen receptor (CAR) T-cell therapy has shown promising early signals of activity in treating select patients with brain tumors^1^. ACT is a type of immunotherapy that uses a patient’s own immune cells to help fight disease, such as cancer. CAR T cells are patient-isolated T cells genetically engineered ex vivo to regain cancer-fighting properties. The efficacy of CAR T for solid tumor types, including brain tumors, remains elusive. However, the approach holds promise as evidenced by (i) responses in small series of brain tumor patients^2,3^; (ii) success of CAR T in B cell neoplasm settings in which six therapies have been FDA approved^4^; and (iii) recent FDA approval of a similar cell therapy approach for advanced melanoma^5^. The autologous in vitro- expanded tumor-infiltrating lymphocyte (TIL) lifileucel was approved by the FDA in 2024 as the first TIL therapy to treat cancer confirming the future use of TILs in mainstream practice. There are also promising TIL early efficacy data in lung cancer^6^, but these findings are yet to be replicated in immunologically “cold” tumors such as glioma^5^. Existing ACT modalities have shown limited efficacy against many brain tumors^7^. This challenge may stem from various mechanistic hurdles, including the scarcity of the brain tumor microenvironment (TME), impaired T cell trafficking to the tumor, downregulated checkpoint molecule expression, tumor heterogeneity, immunosuppressive TME, and lack of tumor antigen presentation^4^ (Fig. 1a).

Several ACT modalities are currently in trial for central nervous system (CNS) tumors, mainly recurrent glioblastoma, with mixed results. Given the heterogeneity of glioblastoma and CNS tumors, most successful trials are targeting multiple tumor antigens with CARs. For example, one clinical study saw no clinical efficacy in recurrent glioblastoma patients cotreated with CAR T-cell therapy targeting epidermal growth factor receptor (EGFR) III and the anti-PD1 antibody pembrolizumab^8^. However, an early phase I clinical trial for recurrent glioblastoma saw success in CAR T cells engineered to target both EGFR III and wildtype EGFR^9^. All patients saw tumor regression, though two of the three patients eventually showed reoccurrence^9^. Similarly, a study using CAR T-cells targeting both EGFR and IL13Rα2 saw regression in 8 of 13 patients with measurable disease at the time of infusion^10^. One confirmed partial response by Modified Response Assessment in Neuro-Oncology criteria was observed^10^. However, patients in both studies exhibited grade 3 adverse events, including neurotoxicity, encephalopathy, and fatigue^9,10^. Further trials of CAR T treatments for brain tumors show limited efficacy and are summarized in review^11^. These inconsistent results and small sample sizes highlight the need for further investigation in CAR therapies for brain tumors.

Radiation therapy (RT) is the standard treatment of brain tumors, offering an opportunity to combine with ACT. The majority of brain tumor patients are candidates for RT with curative or palliative intent^12^. However, the effects of RT on the tumor immune microenvironment are complex. Many of these processes occur acutely after each fraction of RT in a time-limited fashion. These include stimulation of antigen presentation, induction of cytokine release by neoplastic cells and resident immune cells, downregulation of checkpoint protein expression on tumor and immune cells, and attraction of lymphocytes to the tumor site^13–15^. On the other hand, a major obstacle is that many immune cells are radiosensitive and undergo cell death upon entering the RT field^16–19^, hampering the ability of RT to stimulate engineered immune cells or other adaptive immune responses^13,14^ (Fig. 1b). It stands to reason that maximal immune stimulation occurs as tumors are first exposed to RT during the initial fractions of definitive, fractionated RT. Yet this is the precise time when RT is likely killing any CAR T cells or lymphocytes that may enter the radiation field to sample antigens and elicit adaptive immune responses.

Approaches to increase radioresistance of immune cells may hold promise for enhancing ACT efficacy. Increasing radioresistance could overcome the major challenge to combining ACT with RT, which is that many immune cells are highly radiosensitive and die upon entering the radiation field. Engineering radioprotected immune cells could address a critical barrier to the effective combination of ACT with RT (Fig. 1c). The concept of “radioprotecting” T cells for cell therapy has seen limited exploration. Notably, a recent study reported the overexpression of superoxide dismutase 2 (SOD2) in CAR T cells, which yielded promising results in a preclinical model involving head and neck cancer^20^. However, SOD2’s radioprotective effects may be modest^21,22^. HeLa cells with SOD2 overexpression still showed signs of DNA damage and reactive oxygen species (ROS) buildup after treatment with 5 Gy^21^, emphasizing the need for a broader investigation into various candidate genes and the potential of potent radioprotectants derived from extremophiles. Thus, there is an unmet need to identify methods to protect immune cells from RT-induced death to optimize the immune-stimulatory benefits of RT. There is also a pressing need for a deeper understanding of the molecular pathways involved in radioresistance, which could significantly enhance the efficacy of ACT when combined with RT.

Extremophiles, organisms that thrive in extreme environments like high radiation, temperature, or desiccation, possess unique molecular mechanisms to safeguard cellular integrity, making their proteins attractive for radioprotective applications in cell therapy. For instance, the tardigrade-derived Damage Suppressor (Dsup) protein binds to nucleosomes, reducing hydroxyl radical-induced DNA damage and double-strand breaks (DSBs), significantly enhancing cell survival under ionizing radiation without impairing normal functions^23,24^. Notably, local Dsup mRNA nanoparticle delivery has been shown to effectively radioprotect nearby healthy tissue in mice undergoing RT for orthotopic oral cancer^25^. Other promising proteins include PprI from *Deinococcus radiodurans*, which activates DNA repair and reduces apoptosis^26^; TRID1 from tardigrades, which promotes DNA repair through phase separation and repair machinery recruitment^27^; and SASP from *Bacillus subtilis*, which binds to DNA to provide a robust protective shield^28^.

Here we review the literature that could provide insights on approaches to radioprotect immune cells to stimulate ACT for brain tumors. We delineate knowledge gaps and opportunities to advance immune-cell radioprotectors and rationally combine ACT with RT. We highlight extremophile organisms as an intriguing source of radioprotective molecules that could be applied to human immune cells. A variety of approaches to screen for ideal radioprotectors are considered. Safety and ACT manufacturing considerations are evaluated. Finally, we consider different clinical situations in which clinical trials for radioprotected ACT and RT combination treatments could be deployed. Along with this, we review considerations for clinical trial design, ACT administration, rational sequencing of ACT and RT delivery, and selection of RT fractionation schemes.

### Immune-cell types used for ACT and their radiation sensitivity

**Fig. 2:**
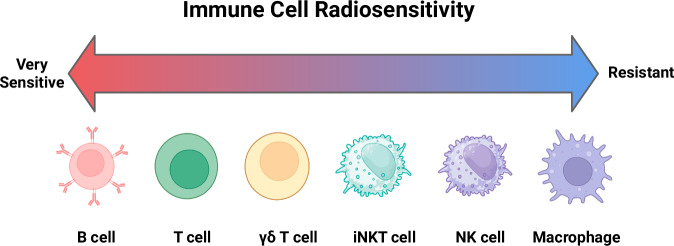
Baseline radiosensitivity of cell types used for ACT. This figure summarizes the inherent radiosensitivity and other effects of irradiation on various immune-cell types commonly used in adoptive cell therapy (ACT). While radiosensitivity is likely highly context-dependent, in general B cells are highly sensitive to radiation and undergo apoptosis at even low doses. T cells are sensitive, with sublethal doses (<2 Gy) causing activation, while higher doses (>2 Gy) induce apoptosis. γδ T cells are relatively resistant to radiation and maintain their cytotoxic functions. Invariant natural killer T cells (iNKT cells) exhibit moderate resistance to radiation. Natural killer (NK) cells and macrophages are relatively radioresistant.

Several immune-cell types have been explored for ACT, including T cells, natural killer (NK) cells, macrophages, and more recently, unconventional lymphocytes such as γδ T cells and invariant natural killer T (iNKT) cells (Fig. 2)^29–31^. Each immune-cell type offers unique advantages in terms of tumor recognition, cytotoxicity, and persistence. Understanding their role within the tumor microenvironment and their radiosensitivity is crucial for optimizing radioprotected ACT in combination with radiotherapy (RT), a standard cancer treatment for brain tumors.

A general theme for all immune-cell types is that RT may stimulate responses at low doses but leads to cell death or decreased responsiveness at higher doses^32^. Patients undergoing standard-of-care RT for glioblastoma, the most aggressive and malignant primary brain tumor, experience worsening lymphopenia (lymphocyte depletion) during RT^17^. The risk of lymphopenia is related to the volume of tissue being irradiated^18,19^. While concurrent chemotherapy is a risk factor, RT alone likely induces lymphopenia via distinct mechanisms^18,19^. Therefore, methods to modulate the radiosensitivity of lymphocytes may be of interest to decrease the risk of lymphopenia and maximize the efficacy of ACT. Understanding the exact radiosensitivity of lymphocytes, however, is imperfect as many experimental methods and results differ, as shown in review^33^. Additionally, some research suggests that sex and age can affect the radiosensitivity of lymphocytes^33^.

#### T lymphocytes

T cells, particularly T cell receptor (TCR)-transgenic T cells and chimeric antigen receptor (CAR) T cells, have been at the forefront of ACT for cancer. CAR T cells, genetically engineered to express receptors targeting tumor antigens, have shown remarkable efficacy in hematologic malignancies^34^. Essentially all FDA approvals for ACT anticancer therapies utilized T cells, including six approvals for CAR T cell infusions and an approval for tumor-infiltrating lymphocytes for advanced melanoma patients^5,34^.

T cells are sensitive to ionizing radiation, which can impair their proliferation and function at high doses. In particular, the D10 (dose required to reduce surviving fraction to 10%) for CD4+ and CD8+T cells is ~3 Gray (Gy)^16^, 20-fold lower than a standard RT dose of 60 Gy. T cells are killed by direct and indirect DNA damage in radiation fields^16^. Fluorescence tracking demonstrates T cell recirculation is transiently impaired by radiation therapy to the tumor^35^. Prolonged or high-dose radiation exposure can induce T cell exhaustion, reduce cytokine production (e.g., interferon-gamma), and increase the expression of inhibitory receptors such as PD-1^36–38^.

Of note, sub-lethal doses of radiation (typically <2 Gy) can enhance T cell activation and recruitment to tumors^39^. Radiation induces the release of neoantigens and pro-inflammatory cytokines, improving T cell recognition of tumor cells^40,41^. Additionally, RT can upregulate major histocompatibility complex (MHC) molecules and death receptors on cancer cells, enhancing T cell-mediated cytotoxicity^42^. Some studies also suggest that low-dose RT enhances the trafficking of T cells into the tumor microenvironment (TME) by modulating the expression of chemokines like CXCL9 and CXCL10^43^.

#### Natural killer (NK) cells

NK cells are innate immune effectors that recognize and kill tumor cells independently of antigen presentation, through mechanisms such as recognition of downregulated MHC class I molecules or through activating receptors like NKG2D. NK cells offer an advantage in brain tumors that evade T cell immunity by downregulating MHC molecules. NK cells do not induce graft versus host disease (GvHD) and can therefore be collected from allogenic donor sources or cell lines, positing the potential development of universal, radioprotected CAR-NK cells targeting common tumor antigens^44^.

NK cells are generally more resistant to radiation than T cells^45,46^. Studies show that NK cell cytotoxicity remains functional at moderate doses of radiation, making them ideal candidates for combination with RT^45^. Some research suggests that fractionation, in comparison to single, large-dose RT, may improve the cytotoxicity and expansion of NK cells^47^. In cancers such as prostate cancer^48^, non-small cell lung cancer^49^, and hepatocellular carcinoma^50^, increased levels of NK cells in the blood have been observed following RT.

Radiation can induce the upregulation of stress ligands (e.g., MICA/B, ULBP1-6) on tumor cells, enhancing NK cell recognition and killing^51^. However, high doses of radiation (>8 Gy) can impair NK cell proliferation and effector functions, including degranulation and cytokine production^46^. Additionally, radiation can alter the expression of NK cell ligands on tumor cells, either enhancing or diminishing NK cell cytotoxicity depending on the radiation dose and tumor type^51^. Interestingly, pre-treatment of the tumor site with low-dose radiation has been shown to prime the tumor for NK cell-mediated lysis, suggesting a synergistic effect when combined with NK-based ACT^52^. This makes NK cells a promising candidate for combinatory therapies involving radiation.

#### Macrophages

Macrophages play a dual role in cancer, either promoting tumor progression (M2-like macrophages) or mediating tumor destruction (M1-like macrophages). Adoptive transfer of macrophages reprogrammed toward an M1 phenotype is an emerging strategy in ACT^53^. These macrophages can be engineered to enhance their phagocytic activity against cancer cells or to produce pro-inflammatory cytokines within the TME.

Macrophages are relatively radioresistant compared to T and NK cells^54^. Low to moderate doses of radiation (≤2 Gy) can induce polarization of macrophages toward an M1 phenotype, promoting anti-tumor activity^55,56^. Radiation enhances macrophage-mediated phagocytosis by upregulating “eat me” signals (e.g., calreticulin) on tumor cells and increasing the production of inflammatory cytokines such as TNF-α and IL-12^57,58^. However, macrophages exposed to high-dose radiation (>2 Gy) may undergo apoptosis or shift toward an M2-like immunosuppressive phenotype, supporting tumor growth and immune evasion^58^. Radiation also affects the recruitment of macrophages into the TME by inducing the expression of macrophage-attracting chemokines, such as CCL2^59^. This can lead to the infiltration of both pro-tumorigenic and anti-tumor macrophages^59^, highlighting the complexity of their role in radiation-enhanced immune responses.

#### γδ T cells

γδ T cells represent a small subset of T cells that recognize non-peptide antigens and exhibit MHC-independent tumor recognition^60^. They have garnered interest for ACT due to their broad tumor specificity and cytotoxic potential^30^. γδ T cells may be useful for pediatric brain tumors, which have lower mutational loads^61^.

γδ T cells are relatively radioresistant and maintain their cytotoxic functions even after moderate doses of radiation^62,63^. Radiation-induced stress ligands on tumor cells, such as NKG2D ligands, enhance the recognition and killing of tumor cells by γδ T cells^51^. Moreover, γδ T cells can proliferate and produce cytokines such as IFN-γ in irradiated tumors, further promoting anti-tumor immunity^64^. The combination of γδ T cell-based ACT with low-dose RT has shown promise in preclinical studies, as radiation not only primes tumors for γδ T cell recognition but also enhances the local recruitment of these cells^51,59,65^.

#### Invariant natural killer T (iNKT) cells

Invariant natural killer T (iNKT) cells are a subset of T cells that bridge innate and adaptive immunity by recognizing glycolipid antigens presented by CD1d molecules^66^. Their ability to produce large amounts of cytokines, such as IFN-γ and IL-4, makes them potent activators of anti-tumor immune responses^66^.

iNKT cells show moderate sensitivity to radiation^67^. Relatively little is known about the impact of radiation therapy on iNKT activity with inconsistent findings in the literature, with some proposing that radiation decreases iNKT anti-tumor activity^67,68^. However, iNKT remain an intriguing target for ACT because they do not induce GvHD, similar to NK cells^69^. iNKT cells also have two target ligands: the natural CD1d ligand and CAR-targeted antigen^69^. CD1d is expressed in several brain tumors, including glioblastoma^70^ and medulloblastoma^71^. Radiation also upregulates the expression of intercellular adhesion molecule-1 (ICAM-1), which is expressed in some gliomas and binds to LFA-1 on the surface of iNKT cells^72–74^. However, like other immune cells, high-dose radiation can reduce iNKT cell viability and function^67^, emphasizing the need for dose optimization when combining iNKT cell ACT with RT.

**Fig. 3:**
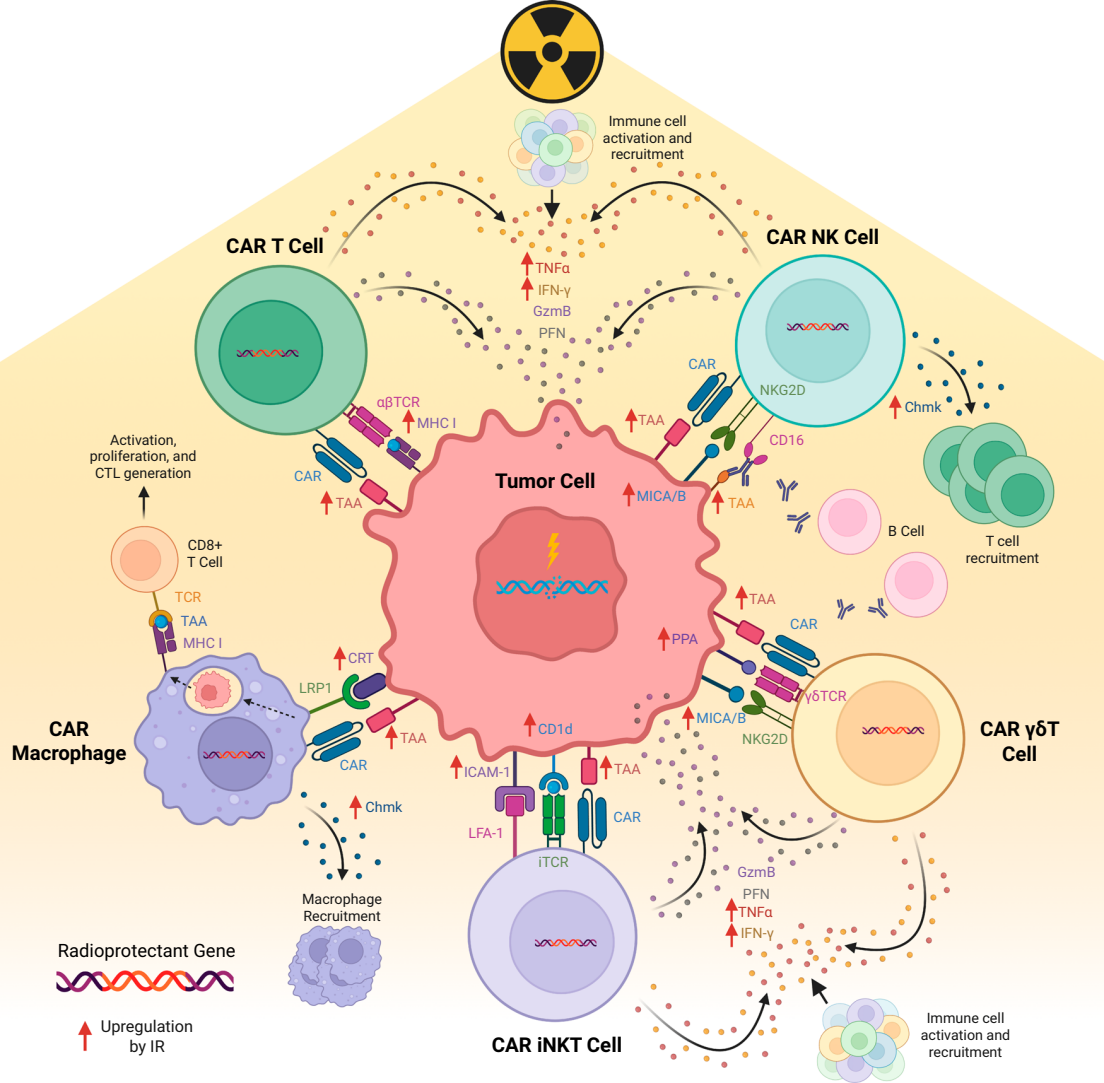
Anti-tumor effects of radiation on CAR immune cells. This figure summarizes the anti-tumor impacts of radiation on CAR T, NK, γδ, iNKT, and macrophage cells. Radiation may illicit an immune response via a variety of mechanisms, including upregulation of tumor antigen presentation and increased cytokine release. TAA: tumor associated antigen; CAR: chimeric antigen receptor; GzmB: granzyme B; PFN: perforin; PPA: phosphoantigen; LRP1: low-density lipoprotein receptor 1; MHC I: major histone compatibility complex I; IFNγ: interferon gamma; TNFα: tumor necrosis factor alpha; Chmk: chemokines; CRT: calreticulin; CTL: cytotoxicity T lymphocyte; CD: cluster of differentiation; MICA/B: MHC class I chain-related protein A and B; TCR: T cell receptor; iTCR: invariant T cell receptor.

Each immune-cell type utilized in ACT for cancer presents distinct advantages and limitations regarding radiation sensitivity. T cells, NK cells, macrophages, γδ T cells, and iNKT cells all exhibit varied responses to radiation, which can be leveraged to enhance their anti-tumor efficacy in combination with radiotherapy. Figure 3 shows each unique CAR cell type and how radiation may increase the anti-tumor response. A strategic combination of ACT with radiotherapy holds significant promise for improving clinical outcomes in cancer treatment, but careful consideration of radiation dosing is critical to maximize synergistic effects while minimizing damage to immune effector cells.

### ACT and chemotherapy

While we propose radioprotected ACT in combination with standard of care RT, many oncologic treatment plans also involve chemotherapies. Radioprotecting gene candidates that modulate DNA damage responses and cell cycle arrest may also decrease the chemosensitivity of the immune-cell^75^. However, chemotherapeutics that preferentially target immune-cell subtypes may negatively affect CAR T efficacy^75^. If ACT must be administered with chemotherapy, researchers may consider engineered expression of chemo-resistance proteins^75^. Modifying cell cycle and DNA damage proteins should be viewed with caution and suicide genes should be added in case of unexpected proliferation (See “Radioprotector safety considerations”)^76^. Of note, some chemotherapeutics may have an immuo-stimulatory effect on the TME that may improve CAR T cell trafficking to the tumor. For example, CAR T cell trafficking to the TME improved in mice preconditioned with temozolomide, a standard chemotherapeutic for glioblastoma, which was linked to decreased regulatory T cell populations in the TME^77^.

### Mechanisms of radioprotection

**Fig. 4:**
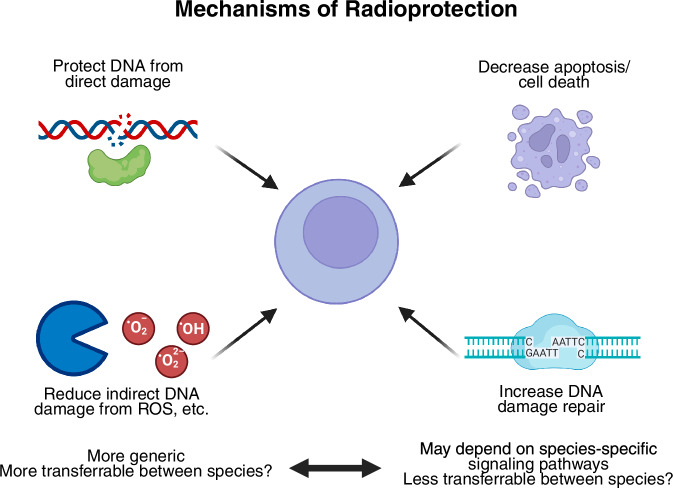
Mechanisms of radioprotection. Radioprotective agents mitigate radiation-induced damage through multiple mechanisms. These include protection of DNA from direct damage, reduction of indirect DNA damage by scavenging reactive oxygen species (ROS), and promotion of DNA damage repair. Additionally, radioprotective strategies may involve decreasing apoptosis and cell death. While reducing ROS is a broadly applicable and potentially safer strategy, inhibiting apoptosis could carry risks, such as increasing the potential for carcinogenesis. Mechanisms related to ROS scavenging are likely to be more transferable between species, while apoptosis modulation may depend on species-specific pathways, raising concerns about their safety and efficacy across different models.

Figure 4 outlines various mechanisms of radioprotection, each with unique implications for efficacy and safety. One mechanism, physically protecting DNA from direct damage by radiation, is likely to be safe, as this simply preserves the integrity of the genetic material without altering cellular processes. Similarly, the reduction of indirect DNA damage caused by reactive oxygen species (ROS) appears to be a generally safe approach. ROS scavenging neutralizes harmful free radicals without disrupting normal cellular functions and may be more universally applicable across species due to its fundamental nature in biology. These strategies aim to protect cells from radiation-induced damage without interfering with critical regulatory processes, making them promising candidates for clinical application.

However, other mechanisms may raise safety concerns. For example, reducing apoptosis could have unintended consequences, such as increasing the risk of carcinogenesis. Apoptosis is a natural defense mechanism that eliminates damaged or potentially cancerous cells. Dampening this process might allow cells with genomic damage to survive and proliferate, potentially leading to tumorigenesis. Similarly, interventions that enhance DNA repair might inadvertently preserve cells with incomplete or improper repair of damaged DNA, increasing the potential for mutations that could lead to cancer. These mechanisms would require careful evaluation in clinical trials to assess the risks and benefits before being considered for non-oncologic applications, such as protecting stem cell allografts during transplantation. It is crucial that any potential radioprotective strategy be scrutinized not only for efficacy but also for long-term safety to prevent unintended consequences like carcinogenesis.

### Extremophiles as a source of radioprotectors

**Fig. 5:**
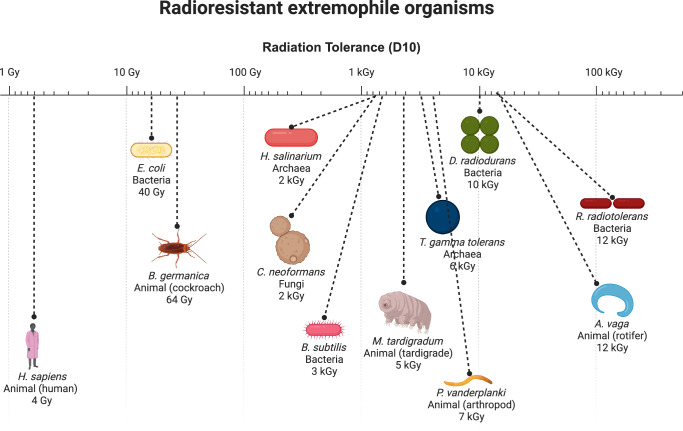
Radioresistant extremophile organisms. Select radioresistant extremophile organisms are shown, compared to humans. Radiation dose in Gray (Gy) for D10 (dose required to kill 90% of organism sample) when available or LD50 (dose required to kill 50% of organism sample) is shown. Note log scale indicating >1000X radioresistance compared to humans for many organisms.

Bridging extremophile biology and immuno-oncology may provide an avenue to radioprotect immune cells for ACT. Extremophiles are organisms that are ultra-tolerant to temperature, pressure, and irradiation extremes. Indeed, a number of bacteria, archaea, fungi, and animal extremophiles exist that can tolerate enormous doses of irradiation (>1000 Gy or 1000 times the human lethal dose, Fig. 5). The genetic basis for this protection is being uncovered by genomic and functional studies, providing a unique opportunity for radioprotection applications. The genetic basis for extremophile radioresistance involves molecules that (i) physically protect DNA from direct damage; (ii) mitigate indirect DNA damage via metabolic activities such as scavenging of reactive oxygen species; (iii) provide improved or redundant DNA damage repair machinery; (iv) alter apoptosis pathways; or (v) modulate cell signal transduction^23,24,28,78–80^. This raises the opportunity to apply emerging extremotolerant technology to T cells to enable therapeutic approaches.

Among the candidates, the Damage Suppressor (Dsup) protein from tardigrades stands out. Tardigrades are water-dwelling, eight-legged micro-animals that can be found everywhere from high-altitude mountaintops to the deep sea. Tardigrades have an extraordinary ability to tolerate immense doses of radiation (>5000 Gy) that would be lethal to most other life forms^23,24^. In the most stress-tolerant Tardigrade species, *Ramazzottius varieornatus*, the Tardigrade-unique damage suppressor (Dsup) protein colocalizes with DNA (nucleosomes in particular) and protects from hydroxyl radicals, protecting cells from radiation-induced DNA damage and cell death^23,24^. Transfection of Dsup into immortalized human cells enabled expression of the Dsup protein with no reduction in cell proliferation^23^. In addition to Dsup, additional radiosensitizers in tardigrades have been explored, including the DOPA (dihydroxyphenylalanine) dioxygenase gene (*DODA1*), tardigrade-specific radiation-induced disordered protein (TRID1), ubiquinol–cytochrome c reductase (bc1) synthesis protein (BCS1), and NADH dehydrogenase (ubiquinone) 1 beta subcomplex subunit 8 protein (NDUFB8)^27^. *DODA1* leads to the production of betalins, a plant pigment with radical-scavenging properties^27^. TRID1 assists with liquid-liquid phase separation and enhances the recruitment of DNA repair protein to the double strand break (DSB) sites^27^. NDUFB8 and BCS1 are non-tardigrade specific proteins part of the mitochondrial respiratory chain complex assembly that are upregulated in tardigrades^27^. These proteins accelerate NAD+ regeneration for PARP1-mediated DNA repair^27^.

Other candidate radioprotectors include *D. radiodurans* PprI, which stimulates DNA repair and radioprotects human cells and mice^26^ and *B. subtilis* small acid soluble protein (SASP) which binds and potently protects DNA^28^. Sulfiredoxin from *C. neoformans* is strongly induced post-irradiation and radioprotects fungi^80^. Also, specific heat shock proteins have been linked to maximal irradiation survival response in the rotifer *R. vega*^81^. Molecules that mitigate direct or indirect DNA damage may be most likely to function in human cells, while molecules that have more complicated functions (such as in damage repair complexes and signaling pathways) may be less likely to do so.

Expression of foreign proteins, or “xenoproteins,” may present its own challenges in the pathway to engineering radioprotected immune cells. Extremophile-derived proteins may cause unexpected effects when expressed in human T cells. Future work could engineer these proteins to mitigate these issues by rationally combining key domains from these proteins with structurally similar human proteins.

### Approaches to identify radioprotectors

**Fig. 6:**
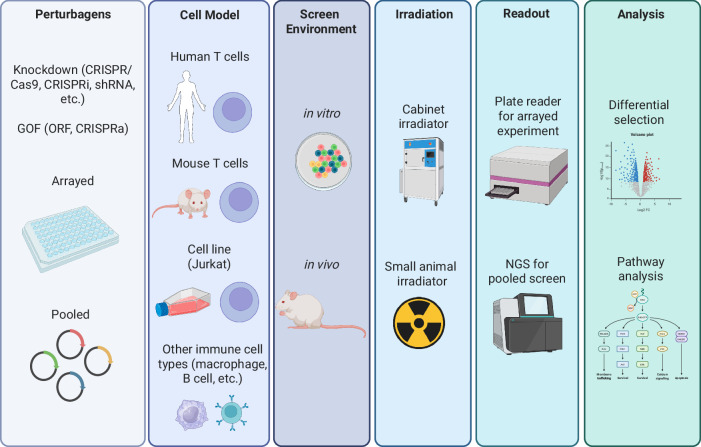
Approaches to screen for immune-cell specific radioprotectors. Various strategies can be employed to identify radioprotectors that specifically target immune cells. High-throughput screening of chemical libraries, genetic screens, and functional assays offer ways to discover agents that selectively shield immune cells from radiation damage. Depending on the type of ACT being studied, screens can be conducted in a range of immune-cell types, from easily manipulated cell lines to more clinically relevant, human-derived cells. Screening can be performed in vitro, which allows for more practical, controlled experiments, or in vivo, where complex biological interactions are better represented. Radiation is applied as needed, using tools like cabinet irradiators for in vitro cultures or small animal irradiators for in vivo studies. Readouts can vary depending on the specific goals of the screen, ranging from simple measurements of cell survival or function post-irradiation to more sophisticated next-generation sequencing (NGS) approaches that assess the selection of different perturbations after exposure. Analytical methods can involve straightforward ranking of top perturbations that improve immune-cell survival or function, as well as deeper molecular pathway analyses to gain mechanistic insight. Radioprotectors are also assessed for their ability to maintain immune-cell functionality, prevent apoptosis, or preserve immune-cell subsets during or after radiation. Validation of hits is critical to confirm radioprotective efficacy and ensure that identified candidates not only protect immune cells but also preserve their therapeutic potential. These methods hold promise for identifying radioprotectors that maintain immune competence in ACT, while ensuring therapeutic safety and efficacy.

Identifying effective radioprotectors requires comprehensive screening strategies that take into account the unique characteristics of human immune cells. One approach is to utilize in vitro genetic screens, which can provide insights into how various candidate genes may confer radioprotection. Both pooled and arrayed screens could be adapted from existing methodologies used to identify costimulatory molecules for CAR T cells^82^, thus prioritizing radioprotective genes for further study. Executing in vivo screens in animal models may be able to capture aspects of therapeutic efficacy in a more complex biological environment. Computational modeling and in silico analyses can also aid in predicting interactions and outcomes, streamlining the discovery process. Considerations for screens are summarized in Fig. 6.

### Logistics of incorporating radioprotectors in cell therapy manufacture

**Fig. 7:**
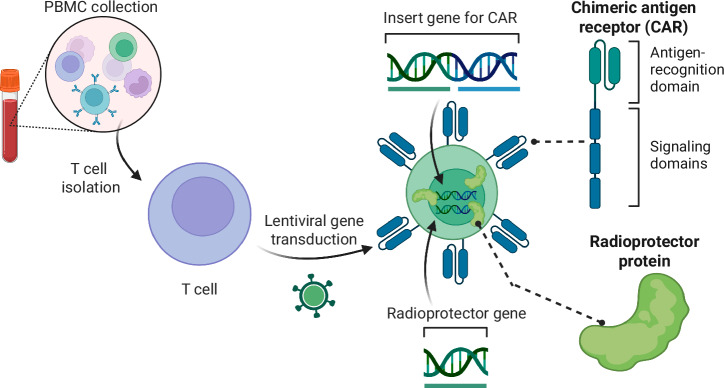
Formulation of radioresistant CAR T cells. CAR T cells are developed from peripheral blood mononuclear cells (PBMCs) which are harvested, expanded, sorted into T cells, and then tranduced with lentiviral vectors to deliver the CAR gene playload. Our proposal will identify the most potent radioprotector genes to deliver in a similar fashion during the CAR T manufacturing process, producing radioresistant CAR T cells.

Integrating radioprotectors into ACT manufacturing processes presents logistical challenges and opportunities. Strategies may involve utilizing the same viral vector for gene delivery or opting for separate vectors (Fig. 7), thereby allowing for a modular design that enables the co-expression of radioprotectors alongside therapeutic genes. This flexibility in vector design will facilitate the tailoring of cell therapies to maximize both therapeutic efficacy and safety.

### RT dose, fractionation, volume considerations, and administration method for combination with ACT

**Fig. 8:**
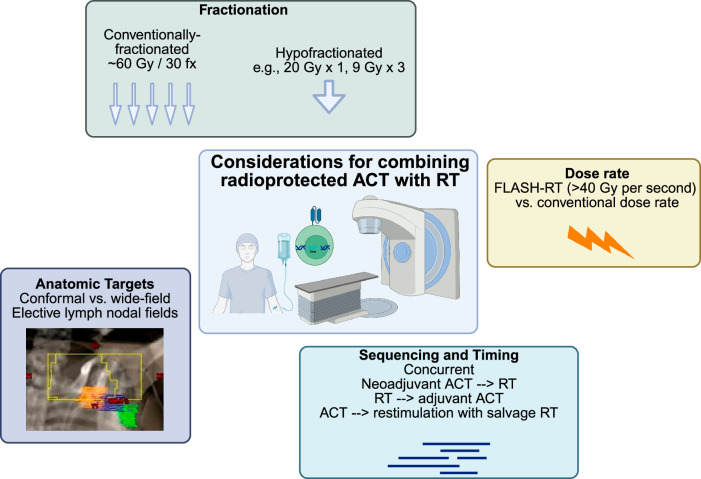
Considerations for RT delivery in combination with ACT. Key factors in optimizing the combination of radiation therapy (RT) with adoptive cell therapy (ACT) are presented. Fractionation options include conventionally-fractionated RT (e.g., 60 Gy in 30 fractions) and hypofractionated RT (e.g., 20 Gy in 1 fraction or 9 Gy in 3 fractions). Dose-rate considerations compare the use of conventional dose rates with FLASH-RT, where doses greater than 40 Gy per second are delivered. Anatomic targets are categorized into conformal vs. wide-field approaches, focusing on elective lymph node fields. Sequencing and timing of treatment strategies include concurrent, neoadjuvant ACT followed by RT, RT followed by adjuvant ACT, and using ACT with salvage RT for restimulation of immune responses.

Combining ACT with radiotherapy offers a potent approach to cancer treatment, but the success of this combination may rely heavily on the careful selection of radiation dose, fractionation, treatment volumes, and administration method to ensure synergy while minimizing damage to immune cells, particularly the infused radioprotected ACT cells. Here, we discuss how different RT regimens and anatomic considerations influence ACT-radiotherapy combinations, and the potential role of radioprotection strategies to preserve ACT cells (Fig. 8).

#### Fractionated RT

Fractionated RT, where a total radiation dose is divided into smaller daily doses, presents an attractive opportunity to combine with radioprotected ACT. Standard fractionation schemes often use daily doses of 1.8–2.0 Gy, which fall near the lethal dose for 50% of T cells (LC50) at ~3 Gy^16^. Fractionated delivery allows immune cells time to recover between doses, reducing the risk of overwhelming damage to ACT cells.

This approach may progressively enhance the recruitment of ACT cells to the irradiated tumor over time^83^. Repeated low-dose exposure can upregulate the expression of stress ligands and increase antigen presentation by tumor cells, thus improving ACT cell recognition and tumor infiltration^84^. Additionally, fractionated RT spares normal tissues, reducing systemic toxicities that could otherwise impair immune responses.

From a logistical perspective, fractionated RT is commonly used in clinical settings for treating brain tumors. Therefore, its widespread clinical use facilitates the integration of ACT without substantial alterations to current RT protocols. Optimizing the timing and sequencing of ACT infusion within a fractionated RT regimen could maximize the beneficial effects of radiation while minimizing immune-cell depletion.

#### Hypofractionated RT

Hypofractionated RT, which delivers higher doses of radiation per fraction (e.g., >8 Gy), poses a different set of challenges and opportunities for ACT combinations. High-dose treatments, such as 20 Gy delivered in a single fraction in stereotactic radiosurgery (SRS) for brain metastases, may result in greater tumor cell death and release of tumor antigens^85,86^. However, the steep increase in radiation dose may overwhelm even radioprotected ACT cells, particularly those sensitive to radiation, such as T cells.

To circumvent the potential for immune-cell depletion, hypofractionated RT may be more effective when administered immediately before ACT infusion rather than concurrently. Pre-irradiation could prime the tumor for ACT by increasing tumor antigenicity and altering the tumor microenvironment to favor immune infiltration without subjecting the infused cells to excessive radiation^85,86^. Careful scheduling of hypofractionated RT prior to ACT infusion may amplify anti-tumor immune responses by using radiation as a priming tool.

#### Anatomic RT field considerations

One critical consideration when combining ACT with RT is the anatomic location and size of the radiation field. While modern RT technologies such as intensity-modulated radiation therapy (IMRT) and stereotactic body radiation therapy (SBRT) allow for precise, conformal delivery of radiation, larger or less targeted radiation fields can pose a risk to immune cells within the lymphatic system and circulating immune cells.

Radiation fields that include lymph node basins or areas of high immune-cell trafficking may reduce the availability of functional immune cells for ACT. Lymph node irradiation may deplete T cells and other immune effectors required for ACT efficacy, which could hinder overall treatment outcomes^87^. Therefore, minimizing the irradiation of at-risk lymphoid structures or employing radioprotective strategies, such as shielding^88^, may help preserve immune functionality in combination therapy.

#### FLASH-RT

An emerging technique in the RT field, FLASH radiotherapy (FLASH-RT), involves ultra-high dose-rate radiation delivery, typically delivered at greater than 40 Gy/s^89^. Studies suggest that FLASH-RT offers the potential to spare normal tissues from the toxicities associated with conventional radiation, while maintaining potent anti-tumor effects^90,91^. The unique biological mechanisms behind FLASH-RT, such as differential oxygen depletion and modulation of the tumor microenvironment, could also offer a new paradigm for combination with ACT^91^. Some research suggests that FLASH-RT can overcome hypoxia-mediated tumor resistance, a hallmark of many brain cancers^92,93^. In glioblastoma, FLASH-RT has also been demonstrated to spare the normal brain from radiation-induced toxicities^94^.

Although still in experimental stages, FLASH-RT may stimulate CAR T cells and other ACT-based therapies in ways that conventional RT cannot. Furthermore, FLASH-RT may alter the TME in a manner that enhances CAR T cell trafficking and persistence, creating a synergistic effect that could amplify anti-tumor efficacy. In murine models of diffuse midline glioma, FLASH-RT led to the upregulation CD4^+^ T cells and genes involved in T-cell activation and trafficking on day 10 following treatment in comparison to conventional radiotherapy^95^. This warrants further investigation into the specific interactions between FLASH-RT and ACT, with an emphasis on understanding the immunological mechanisms involved.

#### Administration

The blood brain barrier and TME pose crucial considerations for the location of ACT administrations. Though ACTs can reach the brain via intravenous injection, other administration methods may increase ACT population within the CNS, possibly decreasing side effects^96^. Intrathecal delivery involves direct injection into the cerebral spinal fluid (CSF), bypassing the blood-brain barrier^96^. Injection can occur into the spine or the ventricles of the brain. While spinal intrathecal delivery, or lumbar punctures, may be useful for single-dose ACT, intraventricular injections, commonly via Ommaya catheters, can be programmed with a pump to deliver repeated doses^96^. Implantable devices may also offer an easier way to obtain CSF samples to confirm immune activation^96^. Intra-tumoral delivery of ACT is also possible via convection enhanced delivery (CED) systems. CED systems form a pressure gradient via a microinjection pump, possibly reaching a larger brain volume^97^. However, the pressure gradient may cause worsening neurotoxicity symptoms, a common side effect in CAR T patients^96^. Further research on the optimal administration method may maximize the response of radioprotected ACT.

### Sequencing of radioprotected ACT and RT

Optimal sequencing of radioprotected ACT and RT is essential to fully leverage the therapeutic potential of these combined modalities. While both ACT and RT are potent treatments on their own, their integration requires careful planning to avoid negative interactions such as radiation-induced immune-cell damage. Incorporating radioprotection strategies into ACT opens new possibilities for safely combining these treatments, potentially allowing for higher radiation doses or more aggressive fractionation schedules. The number of doses should also be considered, as radioprotection may impact the longevity of modified immune cells. Preclinical models and clinical trials are necessary to establish the most effective sequencing protocols.

#### Preclinical studies to inform sequencing

Preclinical studies are particularly crucial for evaluating how the addition of radioprotective strategies influences the interaction between ACT and RT. In these models, the timing of ACT administration relative to RT can be explored, particularly in the context of radioprotection. For instance, studies can assess whether radioprotected ACT cells retain functionality and viability when delivered before, during, or after RT. Additionally, preclinical research can help define the thresholds of RT dose and fractionation at which radioprotected ACT cells are most effective, without suffering significant damage from radiation. By incorporating radioprotective agents into ACT protocols, it may be possible to use higher radiation doses that would otherwise impair T-cell function. This opens new avenues for combination therapies, but the precise sequencing of these treatments will need to be fine-tuned through preclinical work before transitioning into clinical practice.

#### Integrating radioprotected ACT into upfront, fractionated RT for curative-intent treatment

For brain cancers where upfront fractionated RT is the standard of care, such as glioblastoma, integrating radioprotected ACT into curative-intent treatment regimens offers an exciting therapeutic strategy. Fractionated RT typically involves daily radiation doses of around 1.8–2.0 Gy delivered over several weeks, creating a potentially favorable environment for ACT. Radioprotected ACT could be administered early in the course of fractionated RT, allowing T cells to persist and accumulate within the tumor over time.

Because radioprotective strategies could enhance the resilience of ACT cells to low daily doses of radiation, this approach may increase the likelihood of immune cells infiltrating the tumor microenvironment and maintaining functionality throughout the RT course. This might improve the overall therapeutic outcome, especially in brain tumors that are traditionally difficult to treat. In this context, radioprotected ACT cells could be infused early in the RT course and allowed to interact with radiation-induced tumor stress signals and antigen presentation over time. Fractionated RT may also create opportunities for selection of radioprotected ACT cells. While normal immune cells will die throughout fractionated RT, radioprotected ACT cells may not, providing a sustained anti-tumor immune response and reducing the risk of T-cell depletion.

#### Combining salvage RT with radioprotected ACT

For patients undergoing salvage RT, particularly those with recurrent or metastatic cancer, combining radioprotected ACT with higher RT doses or hypofractionated schedules may improve outcomes. In salvage settings, higher RT doses (e.g., 8 Gy per fraction or more) are often required to address treatment-resistant tumors, which can compromise immune-cell viability. The inclusion of radioprotection in ACT protocols allows the possibility of combining these more aggressive RT regimens without overwhelming the immune system.

In these cases, radioprotected ACT could be administered either before or after salvage RT, depending on the patient’s condition and treatment goals. Post-RT administration of radioprotected ACT could allow the immune system to “clean up” any residual tumor cells that survive the high-dose radiation. Alternatively with pre-RT administration of radioprotected ACT, RT could be used to debulk the tumor and prime the tumor microenvironment for subsequent immune attack by radioprotected cells. This combination might offer significant benefits, but the optimal sequencing needs to be explored through preclinical and clinical trials.

#### Re-stimulation of radioprotected ACT with RT

Radiation has the potential to re-stimulate adoptive immune cells that may have become less effective over time, and radioprotected ACT cells could be especially well-suited for this approach. As tumors progress, ACT cells may experience functional exhaustion, particularly in cases of CAR T-cell therapy. RT can induce tumor cell death^85,86^ releasing antigens that could “re-prime” the radioprotected ACT cells, thereby restoring or boosting their anti-tumor activity. In this scenario, radioprotected ACT cells would be better able to withstand the re-stimulation process, maintaining their functionality and viability despite the immunosuppressive effects of radiation. This strategy could be particularly useful in treating brain tumors that exhibit immunoediting, where the immune system drives the selection of tumor cells that evade immune detection^98^. Immunoediting is especially relevant in glioblastoma, which shows poor response to immunotherapies due to tumor herterogeneity^99^. By re-exposing the tumor to immune surveillance following RT, the radioprotected ACT cells could help target these previously elusive tumor cell populations.

### Non-oncologic opportunities

Radioprotective ACT holds promising potential for non-oncologic purposes, such as protecting stem cell allografts during transplantation. One key application could be to reduce the duration and severity of the post-transplant nadir, a period of vulnerability when the patient’s immune system is severely compromised. Typically, patients undergoing stem cell transplantation will receive total body irradiation (TBI) to prep the immune system prior to the transplant^100^. Patients generally receive 12–15 Gy over 3–4 days^100^. TBI acts both cytotoxic and immunosuppressive, creating space in the marrow for new cells and decreasing the likelihood of stem cell rejection^100^. Administering radioprotected ACT prior to radiation could enhance the success and recovery of hematopoietic stem cell transplants, potentially leading to faster immune reconstitution and fewer complications.

Additionally, radioprotected ACT could be explored for specific non-cancerous indications where radiation is necessary but poses risks to healthy cells, such as in certain autoimmune disorders or organ transplants. However, the use of radioprotected ACT for non-oncologic applications would require careful study, as clinical experience builds in oncology. With more research and validation, these innovative approaches could eventually make their way into clinical practice, expanding the therapeutic benefits of radioprotective strategies beyond cancer treatment.

### Alternative radioprotection approaches

Other radioprotective strategies include endogenous pathway modulation, pharmacological agents, physical shields, and cellular engineering, each with distinct limitations. Overexpressing human antioxidants like SOD2 reduces oxidative stress but offers limited protection against high radiation doses, while modulating the p53 pathway can prevent apoptosis but risks preserving genetically unstable cells. Pharmacological options like FDA-approved amifostine provide nonspecific ROS scavenging unsuitable for immune cells, and synthetic molecules such as Mn porphyrins lack DNA repair capabilities. Physical approaches, such as nanoparticle shields, can protect cells but add manufacturing complexity and may hinder cell trafficking. Cellular engineering through CRISPR offers precise genetic modifications for radiation resistance, though extensive screening is needed to avoid adverse effects, while leveraging radiation-resistant stem cell-derived immune cells faces scalability challenges.

### In vivo delivery of CARs and radioprotector genes

The workflow for manufacturing CAR T cells ex vivo for specific patients is long and costly. In vivo delivery proposes the administration of CAR gene or protein payloads enveloped in viral vectors or nanoparticles^101^. Relevant immune-cell-targeting ligands are fused to the viral envelope protein to ensure the proper cells receive the CAR^101^. Lentivirus, retrovirus, and adeno-associated virus have been used in mice to deliver CAR payloads with equivalent efficacy in controlling tumor growth to ex vivo CAR delivery^101^. Nanocarriers delivering mRNA, plasmid, and protein have also been used^101^. In vivo delivery methods may also be helpful for radioprotector gene candidates. For example, delivery of viral vectors encoding a CAR and radioprotector gene could eliminate the need for ex vivo processing. In vivo delivery is still developing and requires careful control to limit off-target effects while maximizing immune-cell transduction efficiency^101^.

### Future clinical trial designs

**Fig. 9:**
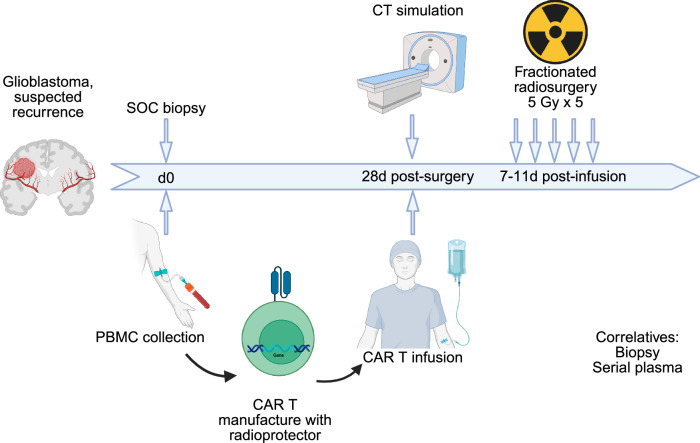
Concept for clinical trial of radioprotected CAR T and RT. Patients with suspected glioma are recruited to the trial. Patients undergo standard-of-care surgical resection followed by radiation therapy. PBMCs are collected and manufactured into CAR T cells including the radioprotector(s) identified here. CAR T cells are re-infused by day 56, providing time for CAR manufacturing but delivering CAR T early in the RT course when immunogenicity may be highest. Concurrent standard-of-care temozolomide may be given or omitted depending on rapid tumor molecular analysis.

Clinical trial design for combining radiation therapy (RT) and radioprotected ACT must thoughtfully consider the optimal sequencing of these treatments to maximize therapeutic efficacy. This involves determining the timing of RT relative to ACT, as well as establishing parameters such as the appropriate ACT dosage, RT fractionation schedules, and dosing to ensure synergistic effects while minimizing toxicity. Additionally, the use of steroids in conjunction with these therapies should be evaluated, as they can impact immune function and treatment outcomes^102^. Incorporating correlative studies into trial designs will enable researchers to explore the underlying biological mechanisms at play, facilitating a better understanding of how these therapies interact. Moreover, identifying and validating biomarkers of response could provide critical insights, allowing for early outcome readouts that guide subsequent treatment decisions and adjustments, ultimately enhancing the overall effectiveness of the combined approach. A schematic for a potential clinical trial of radioprotected CAR T in combination with conventionally-fractionated RT for newly-diagnosed glioblastoma brain tumor patients is outlined in Fig. 9.

### Radioprotector safety considerations

Safety is a paramount consideration when integrating radioprotectors into ACT. First and foremost, the potential for radioprotectors to influence apoptosis or cell cycle pathways raises concerns regarding carcinogenesis. Alterations in these critical regulatory mechanisms may inadvertently promote tumorigenesis, underscoring the necessity for thorough preclinical evaluation and long-term monitoring of patients receiving such therapies. Also, any approach involving the expression of foreign radioprotective proteins must be scrutinized for unforeseen immunogenic effects. The introduction of xenoproteins may elicit immune responses that could compromise the safety and effectiveness of the therapy. Another concern is that CAR T cells expressing genes that modulate the cell cycle or cell senescence could become immortal, potentially leading to rapid cell division, autoimmunity, and/or T cell malignancies. For this reason, researchers should consider integrating suicide genes, such as caspase 9^103^. Suicide genes may also be helpful in case of severe side-effects following CAR T cell infusions, such as cytokine release syndrome^103^. Ideal suicide genes could be activated by a biologically inert, bioavailable antibody^103^.

CAR T trials should also be planned with especially close monitoring and planning for patient safety. Moreover, CAR T cell therapy is known to elicit severe immune reactions, the most notable being neurotoxicity, which can necessitate hospitalization. The introduction of RT may exacerbate these adverse effects, complicating the clinical picture and heightening the need for vigilant monitoring and trial designs that prioritize patient safety. Establishing clear protocols for assessing and managing toxicities will be essential to mitigate these risks effectively. Additionally, the concurrent use of steroids and IL-1R agonists, such as anakinra, should be carefully planned^104^. While these agents can modulate inflammatory responses, their immunosuppressive effects could potentially undermine the therapeutic efficacy of CAR T cells. Thus, balancing the need for symptom management with the preservation of immune functionality will be crucial. Comprehensive safety evaluations and preclinical studies are necessary to identify and mitigate these risks before advancing to clinical trials. In summary, while the potential benefits of radioprotectors in enhancing ACT are compelling, the associated safety concerns must be addressed through rigorous research, careful clinical trial design, and ongoing patient monitoring to ensure a favorable risk-benefit profile.

### Conclusion

The potential to enhance ACT for brain tumors through the radioprotection of immune cells represents a groundbreaking frontier in cancer treatment. Immune-cell types used in CAR therapies exhibit distinct anti-tumor properties that must be carefully leveraged to maximize therapeutic impact. Insights from extremophile biology, coupled with advancements in radioprotective technologies and robust screening methodologies, offer a unique opportunity to overcome the challenges posed by the immunosuppressive tumor microenvironment in brain cancers. However, introducing novel proteins carries inherent risks, underscoring the necessity for extensive preclinical research to optimize the sequencing of ACT and RT for improved efficacy. Collaborative research efforts will be pivotal in translating these innovations into clinical practice, ultimately advancing outcomes for brain cancer patients. Future work should prioritize high-throughput screening to identify additional extremophile-derived proteins with complementary radioprotective functions, alongside the development of sophisticated delivery systems that ensure stable, efficient expression of these genes in immune cells without impairing function. Finally, rigorous safety and immunogenicity testing, including long-term preclinical evaluation of these proteins in CAR T cells, will be essential to validate therapeutic efficacy while minimizing potential adverse effects.

## Data Availability

No datasets were generated or analysed during the current study.
